# Supplementary material for: CSPP1 stabilizes growing microtubule ends and damaged lattices from the luminal side
Source: J Cell Biol. 2023 Feb 8;222(4):e202208062. doi: 10.1083/jcb.202208062 (PMC9948759; doi:10.1083/jcb.202208062)
Supplement: Table S1 — lists key resources. [file JCB_202208062_TableS1.docx]

**Table S1: Key resources table**

| **REAGENT or RESOURCE** | **SOURCE** | **IDENTIFIER** |
| --- | --- | --- |
| ***Antibodies*** | | |
| Mouse anti-acetylated tubulin | Sigma-Aldrich | Cat# T7451 |
| Purified Mouse Anti-EB1 (Clone 5/EB1) | BD Biosciences | Cat# 610535 |
| Rat anti-tyrosinated α-tubulin (Clone YL1/2) | Abcam | Cat# ab6160 |
| Rabbit anti-alpha tubulin | Proteintech | Cat# 80762-1-RR |
| Rabbit anti-CSPP1 | Proteintech | Cat# 11931-1-AP |
| Goat anti-PCM1 (G-6) | Santa Cruz Biotechnology | Cat# sc-398365 |
| Rabbit anti-PCM1 | Abcam | Cat# ab72443 |
| Rabbit anti-actin | Sigma-Aldrich | Cat# A5060 |
| Goat anti-Mouse IgG (H+L), Highly Cross-Adsorbed, Alexa Fluor 594 | Thermo Fisher Scientific | Cat# A-11032 |
| Goat anti-Rabbit IgG (H+L), Highly Cross-Adsorbed, Alexa Fluor 488 | Thermo Fisher Scientific | Cat# A-11034 |
| Goat Anti-Rat IgG H&L (Alexa Fluor® 405) preadsorbed | Abcam | Cat# ab175673 |
| Donkey anti-Goat IgG (H+L), Cross-Absorbed, Alexa Fluor 568 | Thermo Fisher Scientific | Cat# A-11057 |
| AlexaFluor 488 conjugated AffiPure Donkey-anti-Rabbit IgG | Jackson Immuno Research | Cat# 711-545-152 |
| Cy3 conjugated AffiPure Donkey-anti-Mouse IgG | Jackson Immuno Research | Cat# 711-165-150 |
| AlexaFluor 647 conjugated AffiPure Donkey-anti-Rabbit IgG | Jackson Immuno Research | Cat# 711-605-152 |
| AlexaFluor 647 conjugated AffiPure Donkey-anti-Mouse IgG | Jackson Immuno Research | Cat# 711-605-150 |
| Goat anti-Rabbit peroxidase, conjugated | Jackson Immuno Research | Cat# 111-035-144 |
| Donkey anti-Rabbit IgG (H+L), Highly Cross-Absorbed, Alexa Fluor 488 | Thermo Fisher Scientific | Cat# A-21206 |
| Manually conjugated CF680-GFP-Nanobody | Biotium (CF680-dye) + NanoTag (FluoTag®-X4 anti-GFP) | Cat# 92029 + Cat# N0304 |
| FluoTag®-X4 anti-GFP-Alexa 647 | NanoTag | Cat# N0304-AF647-L |
|  |  |  |
| ***Chemicals, Peptides, and Recombinant Proteins*** | | |
| StrepTactin Sepharose High Performance | GE Healthcare | Cat# 28-9355-99 |
| Polyethyleneimine | Polysciences | Cat# 24765-2 |
| cOmplete^™^, EDTA-free Protease Inhibitor Cocktail | Roche | Cat# 4693116001 |
| Tubulin protein: porcine brain | Cytoskeleton | Cat# T240 |
| Tubulin protein (fluorescent HiLyte 488): porcine brain | Cytoskeleton | Cat# TL488M |
| Tubulin protein (rhodamine): porcine brain | Cytoskeleton | Cat# TL590M |
| Tubulin protein (biotin labeled): porcine brain | Cytoskeleton | Cat# T333P |
| PLL-PEG-biotin | Susos AG, Switserland | PLL(20)-g[3.5]-PEG(2)/PEG(3.4)-biotin(50%) |
| Methyl cellulose, 4000 cp | Sigma-Aldrich | Cat# M0512 |
| GMPCPP | Jena Biosciences | Cat# NU-405L |
| GTP | Sigma-Aldrich | Cat# G8877 |
| Glucose oxidase | Sigma-Aldrich | Cat# G7141 |
| Catalase | Sigma-Aldrich | Cat# C9322 |
| DTT | Sigma-Aldrich | Cat# R0861 |
| k-casein | Sigma-Aldrich | Cat# C0406 |
| Neutravidin | Invitrogen | Cat# A-2666 |
| d-Desthiobiotin | Sigma-Aldrich | Cat# D1411 |
| Taxol | Sigma-Aldrich | Cat# T7402 |
| Vinblastine sulfate salt | Sigma-Aldrich | Cat# V1377 |
| Fchitax-3 | (Diaz et al., 2000) | N/A |
| Glucose Oxidase (GLOX-buffer) | Sigma-Aldrich | Cat# G2133 |
| Catalase (GLOX-buffer) | Sigma-Aldrich | Cat# C40 |
| MEA | Sigma-Aldrich | Cat# M9768 |
| SuperSignal™ West Dura Extended Duration Substrate | Thermo Fisher Scientific | Cat# 34075 |
| StrepII-GFP-CSPP-L full-length (CSPP-L FL) | This study | N/A |
| StrepII-GFP-CSPP-L 295-653^705-1221 (CSPP-S FL) | This study | N/A |
| StrepII-GFP-CSPP-L 295-849 (CSPP-L MTORG) | This study | N/A |
| StrepII-GFP-CSPP-L 295-653^705-849 (CSPP-S MTORG) | This study | N/A |
| StrepII-GFP-CSPP-L 295-653 (H4+L4+H5) | This study | N/A |
| StrepII-GFP-CSPP-L 295-594 (H4+L4) | This study | N/A |
| StrepII-GFP-CSPP-L 295-594+LZ (H4+L4+LZ) | This study | N/A |
| StrepII-GFP-CSPP-L 375-412+(G_4_S)_2_+LZ (H4+LZ) | This study | N/A |
| StrepII-GFP-CSPP-L 375-453+LZ (MTB+LZ) | This study | N/A |
| StrepII-GFP-CSPP-L 375-422^583-653^705-849 (H4+H5+H6) | This study | N/A |
| StrepII-GFP-CSPP-L 375-453^583-653^705-849 (MTB+H5+H6_706-780_) | This study | N/A |
| StrepII-GFP-CSPP-L 375-453^583-653^705-796 (MTB+H5+PD) | This study | N/A |
| StrepII-GFP-CSPP-L 375-453^583-653^705-780 (MTB+H5+H6_706-780_) | This study | N/A |
| StrepII-GFP-CSPP-L 375-453+LZ+705-796 (MTB+LZ+PD) | This study | N/A |
| StrepII-GFP-MCAK | (Aher et al., 2018) |  |
| mCherry-EB3 full-length | (Montenegro Gouveia et al., 2010) | N/A |
| GFP-EB3 full-length | (Montenegro Gouveia et al., 2010) | N/A |
| StrepII-GFP-MAP7 N-terminus | (Hooikaas et al., 2019) | N/A |
| FuGENE6 | Promega | Cat# E2691 |
| SNAP-Surface® Alexa Fluor® 647 | NEB | Cat# S9136S |
| SNAP-Abberior FLUX 680 | Abberior | FX680 |
| Glutaraldehyde | Electron Microscopy Sciences | Cat# #16110 |
| Lacey EM grids | SPI supplies | Cat# 3840G |
| 5 nm gold particles | Sigma-Aldrich | Cat# 808628 |
| Spherical Gold Nanoparticles (200 nm) | Nanopartz | Cat# A11-200-CIT-DIH-1-10 |
|  |  |  |
| ***Experimental Models: Cell Lines*** | | |
| Human: HEK293T | ATCC | CRL-11268 |
| Monkey: COS-7 | ATCC | CRL-1651 |
| Human: hTERT RPE-1 expressing mNG-CSPP-L | (Frikstad et al., 2019) | N/A |
| Human: hTERT RPE-1 expressing mNG-ARL13B | This paper; plasmid kindly given by Kristen Verhey. | N/A |
|  |  |  |
| ***Oligonucleotides*** | | |
| Control siRNA (targeting eGFP, but not mNeonGreen:  ACGUAAACGGCCACAAGUUC | This paper | N/A |
| siGENOME SMARTpool Human PCM1  Target sequence 1: AAACUGAAACAGCGGAUAA  Target sequence 2: GAAAGCAGUUCCUCUAUUG  Target sequence 3: UCACUUAGAUCAAGCAUUA  Target sequence 4: GCAAAUAGAUCAUCAGAAA | This paper | N/A |
|  |  |  |
| ***Recombinant DNA*** | | |
| Human EB3-mCherry | (Stepanova et al., 2003) | N/A |
| Human βIVb-tubulin-mCherry | (Bouchet et al., 2016) | N/A |
| Human StrepII-GFP-MAP7 | (Hooikaas et al., 2019) | N/A |
| StrepII-GFP-CSPP-L full-length (CSPP-L FL) | This study | N/A |
| StrepII-GFP-CSPP-L 375-453+LZ+705-796 (MTB+LZ+PD) | This study | N/A |
| StrepII-GFP-CSPP-L 375-453+LZ (MTB+LZ) | This study | N/A |
| StrepII-GFP-CSPP-L 375-412+(G_4_S)_2_+LZ (H4+LZ) | This study | N/A |
|  |  |  |
| ***Software and Algorithms*** | | |
| ImageJ | NIH | https://imagej.nih.gov/ij/ |
| Axiovision 4.8.2 | Carl Zeiss | N/A |
| softWoRx | GE Healthcare | N/A |
| Metamorph Version 7.8 | Molecular Devices | https://www.moleculardevices.com/products/cellular-imaging-systems/acquisition-and-analysis-software/metamorph-microscopy |
| GraphPad Prism 9 | GraphPad Software | https://www.graphpad.com/scientific-software/prism/ |
| KymoResliceWide plugin | Eugene Katrukha | https://github.com/ekatrukha/KymoResliceWide |
| DoM Utrecht plugin | Eugene Katrukha | https://github.com/ekatrukha/DoM_Utrecht |
| MATLAB | MathWorks | https://www.mathworks.com/ |
| MATLAB code for max intensity fit of EB3 comet and AUC of fitted CSPP-L signal | (Rai et al., 2020) | N/A |
| IMOD v. 4.11 | (Kremer et al., 1996) | https://bio3d.colorado.edu/imod/ |
| EMAN 2, tomoseg | (Chen et al., 2017) | https://cryoem.bcm.edu/cryoem/downloads/view_eman2_versions |
| Python scripts for cryoCARE denoising | (Buchholz et al., 2019; Ogunmolu et al., 2021) | https://github.com/NemoAndrea/cryoCARE-hpc04 |
| MATLAB scripts for analysis of protofilament shapes | This study | https://github.com/ngudimchuk/Process-PFs |
| UCSF Chimera | (Pettersen et al., 2004) | https://www.cgl.ucsf.edu/chimera/download.html |
| MATLAB and Fiji Groovy scripts for MINFLUX Analysis | This study | https://github.com/EMBL-ICLM/microtubule_width_measurement_MINFLUX |
