# Supplementary figures and images for: CSPP1 stabilizes growing microtubule ends and damaged lattices from the luminal side
Source: J Cell Biol. 2023 Feb 8;222(4):e202208062. doi: 10.1083/jcb.202208062 (PMC9948759; doi:10.1083/jcb.202208062)

Figure S1A: SII-GFP-CSPP-L FL

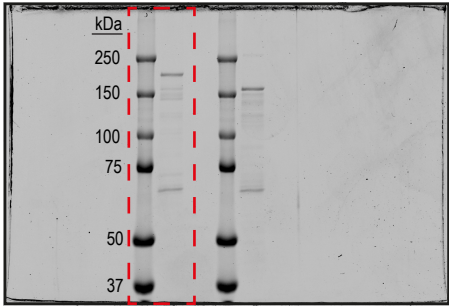

Figure S1G: PCM1

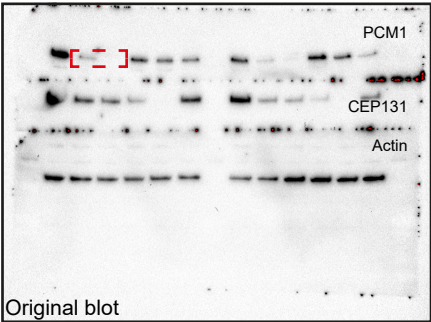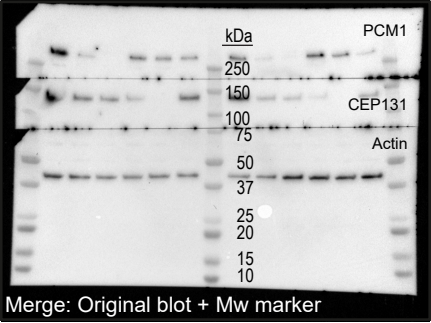

Figure S1G: CSPP1<sup>(1)</sup> + Actin<sup>(2)</sup>

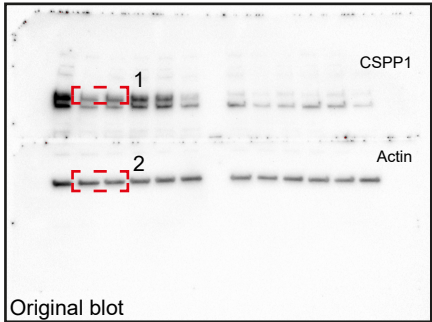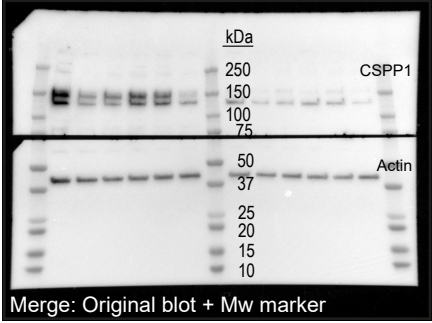

Supplement: SourceData FS1 — is the source file for Fig. S1. [file JCB_202208062_SourceDataFS1.pdf]
