# Supplementary material for: CSPP1 stabilizes growing microtubule ends and damaged lattices from the luminal side
Source: J Cell Biol. 2023 Feb 8;222(4):e202208062. doi: 10.1083/jcb.202208062 (PMC9948759; doi:10.1083/jcb.202208062)

van den Berg et al. - SourceDataFS2

A

Figure S2A: SII-GFP-CSPP-S FL

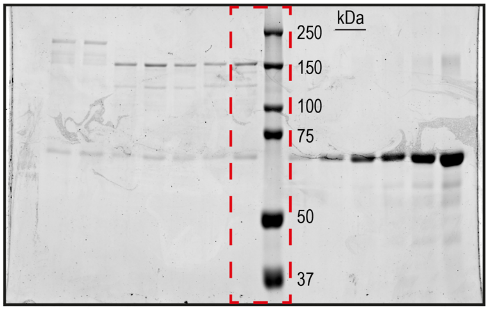

B

Figure S2B: SII-GFP-CSPP-L MTORG

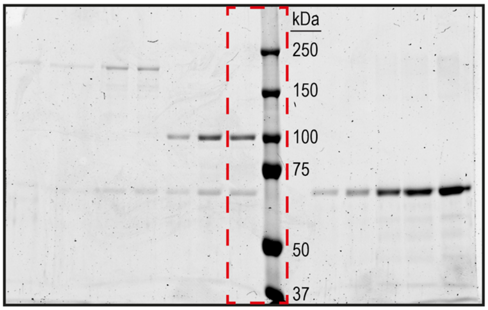

C

Figure S2C: SII-GFP-CSPP-L MTORG

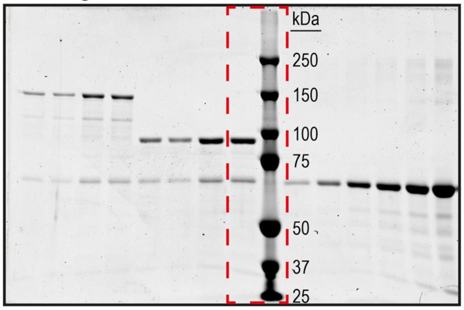

D

Figure S2D: SII-GFP-H4+L4+H5

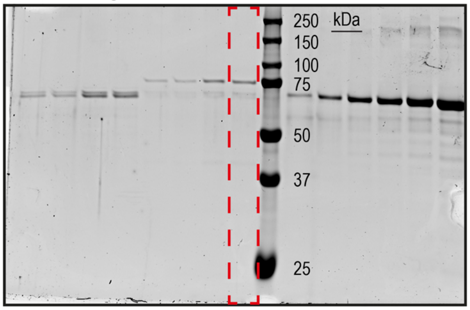

E

Figure S2E: SII-GFP-H4+L4

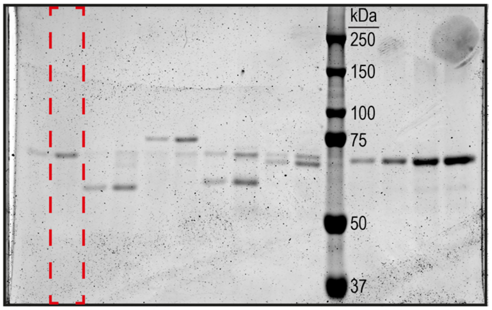

F

Figure S2F: SII-GFP-H4+L4+LZ

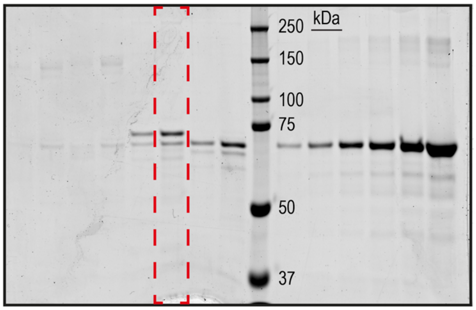

G

Figure S2G: SII-GFP-H4+LZ

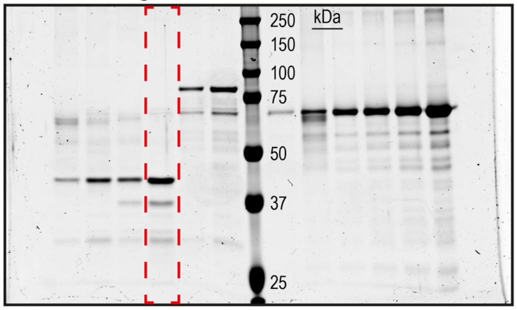

H/M

1:Figure S2H: SII-GFP-MTB+LZ &
2:Figure S2M: SII-GFP-MTB+LZ+PD

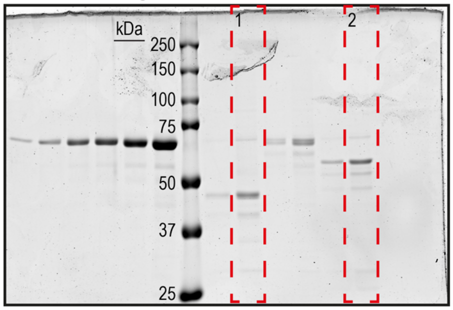

I

Figure S2I: SII-GFP-H4+H5+H6

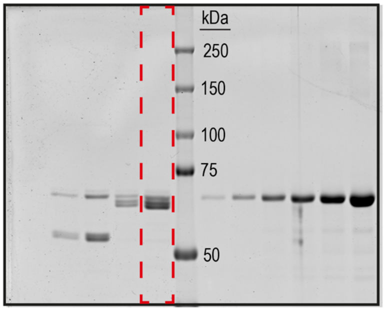

J

Figure S2J: SII-GFP-MTB+H5+H6

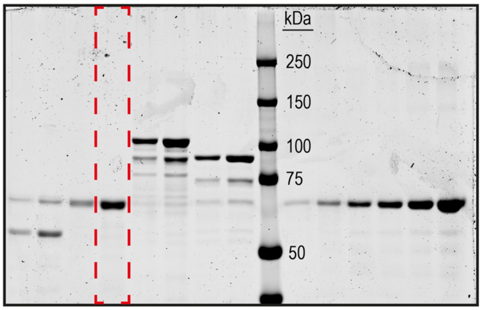

K/L

1:Figure S2K: SII-GFP-MTB+H5+PD
2:Figure S2L: SII-GFP-MTB+H5+H6$_{706-780}$

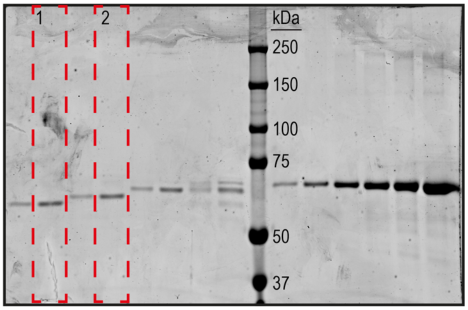

Supplement: SourceData FS2 — is the source file for Fig. S2. [file JCB_202208062_SourceDataFS2.pdf]
